# The Strange Case of the Armored Scale Insect and Its Bacteriome

**DOI:** 10.1371/journal.pbio.0020043

**Published:** 2004-03-16

**Authors:** Benjamin B Normark

## Abstract

Armored scale insects are unusual in that a part of their bodies is genetically distinct from the rest. This extraordinary phenomenon challenges the notion of identity

I am a clone. That is, I am a colony of cells that developed from a single fertilized egg cell. Most animals are clones like me. It is a slight oversimplification to say that all of an animal's cells are genetically identical to each other. Some cells have mutations. In mammals, some cells (red blood cells) lack a nuclear genome entirely. Some cells have viruses—and when it's in a cell, a virus is basically a gene—that other cells lack. But a typical animal is a clone in the sense that all its cells arise from that single fertilized egg cell.

Not all animals, however, are clones. Sometimes two tiny embryos developing inside their mother will fuse together into a single embryo and continue developing. The resulting animal is not a clone, but a chimera: a conglomeration of two different cell lineages into a single organism. Some species of monkeys (marmosets) typically have chimeric blood, from having shared a blood supply with a twin in utero (Haig 1999), and rare cases of accidental chimerism are known from many animal species (Tremblay and Caltagirone 1973; van Dijk et al. 1996). In marine invertebrates, chimeric individuals often arise from the fusion of individuals later in development (Buss 1987). Here I want to draw attention to a remarkable form of chimerism found in armored scale insects. These insects (Figure 1) always develop not from a single fertilized egg but from two genetically different cells. One of these cells develops into a special organ (the bacteriome, which houses symbiotic bacteria) that has a nuclear genome different from that found in the rest of the body.

**Figure 1:**
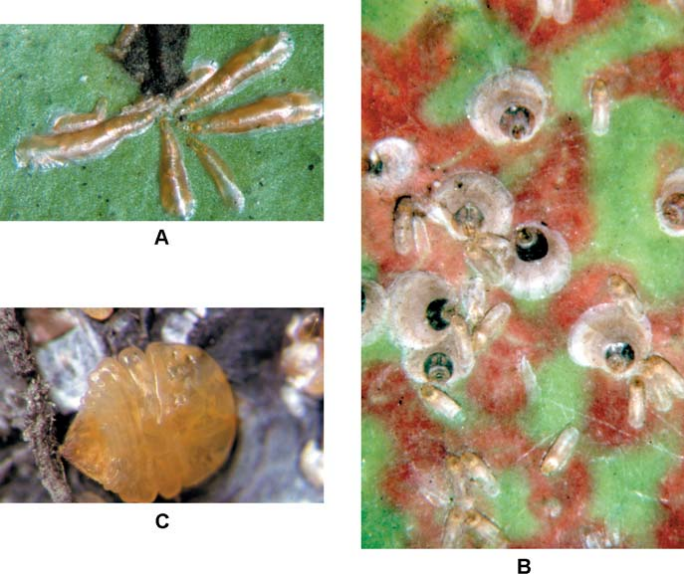
Armored Scale Insects (A) Lepidosaphes gloverii, adult females. (B) Parlatoria oleae, adult females (circular, with dark spot) and immatures (oblong). (C) Quadraspidiotus juglansregiae, adult female with waxy scale cover removed. (Photographs by Raymond J. Gill, © 2003 California Department of Food and Agriculture, published here under the terms of the Creative Commons Attribution License.)

Obligate chimerism—the presence of two genetically distinct cell lineages in every individual at each life stage—is found in a few families of scale insects, but nowhere else in nature. The avid naturalist wants to understand this sort of deep oddity for its own sake, but such understanding might have broader implications as well. For instance, although humans are not usually chimeras, we do have a quasi-chimeric phase in our life cycle: pregnancy. Some diseases of pregnancy are apparently due to conflicts between the genetically nonidentical tissues of mother and fetus (Haig 2002). And the main things that humans eat are also quasi-chimeras: the seeds of flowering plants. In a grain of wheat, for instance, the germ, the endosperm, and the bran have three different nuclear genomes, and the conflicts between them may be similar in some ways to the conflicts seen in human pregnancy (Alleman and Doctor 2000; Santiago and Goodrich 2000). Ultimately, we might learn something about the general principles of conflict and cooperation between maternal and embryonic tissues that govern these cases if we can understand the uniquely stable and intimate chimerism of armored scale insects.

## Two Different Cell Lineages

**Figure 2:**
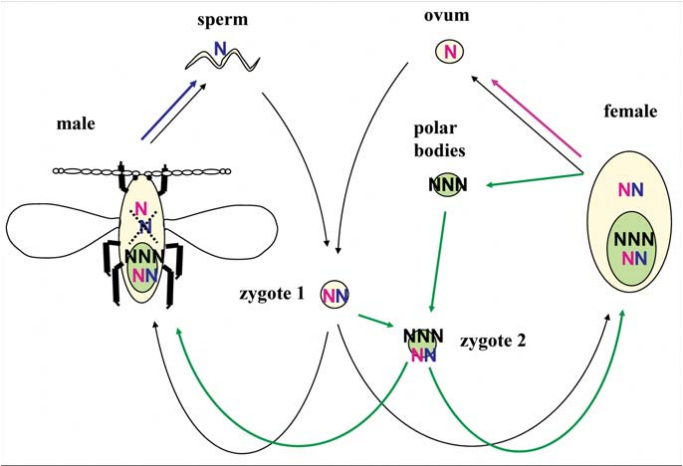
Schematic Diagram of the Genetic System of a Diaspidid Scale Insect Zygote 1 is the fertilized ovum from which all organismal tissues except the bacteriome develop (black arrows). The bacteriome develops from zygote 2 (green arrows). Each haploid genome is represented by an N. A haploid genome may come from the oocyte (pink), sperm (blue), or polar bodies (black). The blue and pink arrows emphasize that the maternal versus paternal identity of a haploid genome is reset (imprinted) in each generation; thus, a male transmits only his maternal genome, but in his offspring the same chromosomes behave as a paternal genome (schematically, the pink N is converted to blue during spermatogenesis).

In all sexual animals and plants, production of an egg cell involves meiosis, the complex cellular process (involving DNA replication, recombination, and two nuclear divisions) whereby one diploid nucleus (with two copies of each chromosome) becomes four genetically different haploid nuclei (each with one copy of each chromosome). Only one of these four haploid nuclei becomes the egg cell (oocyte). In ordinary animals, the other three nuclei (the polar bodies) degenerate—they never divide again and are lost or destroyed—and the oocyte is the single maternal cell that (after fusion with a single paternal cell, the spermatocyte) develops into the embryo. But in armored scales, the polar bodies fuse together into a triploid cell (with three copies of each chromosome), and this triploid cell also winds up in the embryo (Figure 2). The triploid cell derived from the polar bodies fuses with one cell from the embryo to become a pentaploid cell (with five copies of each chromosome). This pentaploid cell then proliferates to form the bacteriome of the embryo (Brown 1965). Each cell in the bacteriome thus contains two copies of the mother's complete genome, in addition to the same haploid paternal genome as the rest of the embryo. In contrast, the rest of the embryo contains just one copy of half of the mother's genome. The apparent function of the bacteriome is to house intracellular bacteria. During embryonic development, bacteria move from the mother's bacteriome into the cells of the embryo's bacteriome. The precise role of the bacteria is not known, but it is thought that they synthesize essential nutrients (Tremblay 1990), as they do in scale insects' close relatives, the aphids (Shigenobu et al. 2000).

Now the story gets even stranger. If the individual is a male, then the genetic difference between his bacteriome and the rest of his tissues becomes even greater as he develops. This is because most armored scale insects have an unusual genetic system called embryonic paternal genome elimination (Herrick and Seger 1999). In males, the paternal genome is completely eliminated from most tissues very early in development—but it is never eliminated from the bacteriome. As a result, most of a male armored scale insect's tissues (including his sperm) have one copy of half of the mother's genome (the same genome as the oocyte from which he developed), but his bacteriome has two complete copies of the mother's genome and also has a paternal genome. Thus, 60% (three of five) of the gene copies in the male's bacteriome are absent from the rest of the male (Figure 2).

## Chimerism and Sibling Rivalry

What could possibly be going on here? Why should scale insects, of all creatures, have obligate chimerism involving activated polar bodies? Essentially, we have no idea, largely because no one has even ventured a serious guess. When the phenomena were discovered, early in the 20th century, the theoretical tools for making sense of them were unavailable. One such tool is W. D. Hamilton's (1964a) theory of inclusive fitness, which holds that the degree of cooperation between two organisms (or tissues) must depend upon their degree of genetic relatedness. But the rise of Hamiltonian thinking coincided with the eclipse of classical cytogenetics in favor of the molecular biology of model organisms, and these remarkable little chimeras have languished in undeserved obscurity. Perhaps merely by looking at them with a modern eye, we can turn up some plausible hypotheses.

Consider the special theoretical difficulty posed by chimerism between tissues derived from the oocyte and those derived from the polar bodies ejected by it during meiosis. Two siblings will typically exhibit some degree of sibling rivalry—their interests are not identical. If an individual were a chimera comprising full-sibling tissues (identical across approximately half of their genomes), there might be conflict between the two nonidentical cell lineages, as there is between the tissues of a mother mammal and her fetus (also identical across half of their genomes) during pregnancy (Haig 2002). This may explain why obligate sibling chimerism never evolves (except perhaps in the very limited case of blood cells between sibling marmosets). But the problem of cooperation between tissues that derive from the oocyte and those that derive from the polar bodies is, if anything, even greater. The oocyte and the polar bodies are *less* closely related than two siblings would be, because the polar bodies are enriched for chromosome regions *not* present in the oocyte.

If there were no crossing over between homologous chromosomes during meiosis, then the first meiotic division would consistently separate the chromosomes derived from the mother's mother from the chromosomes derived from the mother's father, producing two cells that are not related to each other *at all* (or, more precisely, exactly as closely related to each other as the mother's mother was to the mother's father). Crossing over prevents this, creating a mosaic of related and unrelated chromosome regions between the products of the first meiotic division and uncertain relationships between the final four meiotic products. Nonetheless, the consistently depressed relatedness between the oocyte and the polar bodies may help to explain why polar bodies are almost always eliminated—sibling rivalry might be even greater if some siblings were derived from each other's polar bodies.

## Towards an Explanation

So how and why did two families of scale insects tame and domesticate their potentially fractious polar bodies, rather than killing them like normal animals do? There are at least three lines of thinking that seem promising for unraveling this mystery.

### Histological eusociality and relatedness.

There are interesting parallels between, on the one hand, the chimerism seen in armored scale insects and, on the other hand, the eusociality (true sociality) seen in ants and honeybees. In ants and honeybees, sterile individuals (workers) provide nutrition to their potentially fertile siblings. In armored scale insects, a genetically distinct but ultimately sterile cell lineage (the bacteriome) provides nutrition to its potentially fertile “sibling” cell lineage (the rest of the scale insect)—though, of course, polar body-derived cells are “sibling” in a strange special sense. Like ants and bees, armored scale insects are effectively haplodiploid: males transmit only the chromosomes they inherited from their mother, and all of a male's sperm are identical to each other. This “clonality” of sperm boosts the relatedness between sisters, and Hamilton (1964b) pointed out that this high relatedness can explain the high level of cooperation between sisters seen in eusocial ants and honeybees. High levels of cooperation between sisters have since been found in haplodiploid thrips as well (Crespi 1992). It is tempting to speculate that similar explanations can be applied to “histological eusociality” seen in the cooperation between related tissues in scale insects.

This temptation increases when we consider another case from the old cytogenetics literature of apparent histological eusociality, though not of true permanent chimerism. This occurs in a few families of parasitoid wasps (Tremblay and Caltagirone 1973; Strand and Grbic 1997), in which cells derived from polar bodies form a membrane around the yolkless egg that is deposited in the wasp's insect host (which is often a scale insect!). Similar to the worker ant and the scale insect bacteriome, this membrane is thought to mediate nutrition of the developing embryo, and, similar to ants, honeybees, and scale insects, these wasps are haplodiploid.

But although it is tempting to conclude that haplodiploidy plays the same role in promoting histological eusociality as it does in promoting organismal eusociality, the temptation should probably be resisted. In the case of the parasitoid wasps, the polar body-derived tissue has no paternal genome, so the clonality of sperm cannot boost its relatedness to anything. In the case of armored scale insect chimerism, the bacteriome does have a copy of the paternal genome, and that copy is identical to the paternal genome in the rest of the embryo, so in that sense the two tissues do have a high relatedness similar to the high relatedness of full siblings under haplodiploidy. But the scale insect bacteriome gets its copy of the paternal genome directly from the embryo, so the clonality of sperm (the source of elevated relatedness between haplodiploid sisters) apparently has nothing to do with it. Some other explanation is probably needed.

### Maternal interests.

Note that, whatever the relationship between the polar body-derived tissues and the rest of the insect, the polar bodies contain the mother's complete genome. And while your polar bodies may be *less* related to you, on average, than your siblings are, they are *more* related to your siblings (and, of course, to your mother) than they are to you! Perhaps the polar bodies function to somehow enforce some maternal or family interest, nipping in the bud some sibling rivalry that would otherwise suppress the family's fitness. In haplodiploid groups, females are more closely related to their full sisters (with whom they share three-quarters of their gene copies) than to their brothers (with whom they share only one-quarter of their gene copies). So if there is competition between siblings for resources, females are expected to behave more antagonistically towards brothers and more cooperatively towards sisters. In contrast, a female is equally related to a son as she is to a daughter (each carries half of her gene copies). These asymmetries in relatedness lead to struggles in haplodiploid social groups, with a mother seeking to direct more resources towards sons and with sisters seeking to direct more resources away from their brothers and towards each other (Seger and Stubblefield 2002).

It is difficult to see how such a struggle might play itself out in scale insects, which are hardly social insects. The only motile stage in a female's life is the first instar (“crawler”), after which she settles in one spot permanently. The male is slightly more mobile, having a motile (usually winged) adult form. Nonetheless, (1) the low motility of females, and the fact that they live mostly on long-lived woody plants, means that maternal kin may interact over long timescales, as in social insects; (2) some scale insects appear to make relatively sophisticated social decisions about where to settle, settling nearer to (and thus possibly competing more closely with) non-kin than kin (Kasuya 2000); (3) although most scale insects use phloem sap, an almost inexhaustible resource, armored scales use parenchyma tissues (Rosen 1990), which may be locally exhausted, and therefore may compete against neighbors for food. Thus, it is conceivable that females may compete against brothers and sisters for resources, that they may make decisions that affect the intensity of that competition, and that such decisions may have different optima from the perspectives of maternal versus paternal genes. Possibly, the presence of a massive contingent of maternal genes (a double dose of the complete maternal genome) in a nutritionally significant tissue like the bacteriome might somehow affect such decisions in ways favorable to maternal interests. Proximal mechanisms might include effects on signals or perceptions related to relatedness, gender, site quality, or satiety.

Similar manipulation of intersibling interaction might be going on in the case of the parasitoid wasps that have polar body-derived membranes around their eggs. Sometimes these wasps lay a single unfertilized (male) egg and a single fertilized (female) egg into the same host. Both eggs divide to form embryos, which divide into a clone of many embryos. Some of the embryos become sterile “precocious” larvae that can apparently attack other larvae trying to use the same host—including, potentially, their own siblings (Ode and Hunter 2002). Here is a situation in which the polar body genes (in the membranes surrounding the proliferating embryos) might have very different selective optima for levels of between-sibling aggressiveness—and even for rates of development—than the genes in the embryos they surround, and (because they apparently mediate the nutrition of the embryos) they might be able to influence how the embryos develop.

### Gender crypsis.

The endosymbiotic bacteria that dwell in the scale insects' bacteriomes are maternally inherited. Thus, from the perspective of the bacteria, male insects are deadends. Many maternally inherited bacteria have evolved to manipulate the hosts' genetic system for their own advantage. Some bacterial lineages induce parthenogenesis or feminize males. Bacteria may even evolve to suicidally kill male embryos that they find themselves in, if the death of the male frees up resources that his sisters can use (Majerus 2003). In order to do this, bacteria must respond to some cue that indicates the gender of the individual they are in. Potentially, a host could evolve resistance to such manipulation by maternally inherited bacteria by depriving those bacteria of cues indicating gender. In armored scales, the bacteriome has exactly the same genome (two complete copies of the mother's diploid genome and one complete copy of the father's genome) in all full siblings, whether they are male or female, and the same is usually true in mealybugs. This might explain why the bacteriome is the only tissue in which the father's genome remains present and active in both males and females.

## Prospects

Scale insects and their bacteriomes challenge our notion of what an individual is. Is a scale insect's bacteriome a kind of sibling? Is it half sibling, half self? Is it a sterile slave, under control? Is it an extension of the mother, exerting control? In all other organisms, chimeras are temporary and unstable. How have scale insects suppressed the conflicts that normally tear chimeras apart? To approach such questions, we'll have to revive the empirical study of scale insect bacteriomes, combining approaches from recent studies of aphid bacteriomes (Braendle et al. 2003) and of human pregnancy (Haig 2002). We can better understand the nature of genetic conflicts in scale insects by studies of the genetic structure of scale insect populations, together with studies of sex ratio variation and the proximate mechanisms of sex determination. For simplicity, I have described only the most common of the huge variety of very different scale insect genetic systems and modes of bacteriome development (Tremblay 1977, 1990; Nur 1980). This diversity (greater than for the comparable cases of mammalian placentas and flowering-plant endosperms) means there is a huge scope for comparative ecological and genetic studies that could help elucidate general principles. The study of truly strange creatures can tell us what kinds of things are possible. That's why we will be so interested in any life found on another planet and why, in the meantime, we should take a close look at scale insects.
